# Daily Tracking of Glioblastoma Resection Cavity, Cerebral Edema, and Tumor Volume with MRI-Guided Radiation Therapy

**DOI:** 10.7759/cureus.2346

**Published:** 2018-03-19

**Authors:** Shahil Mehta, Shefali R Gajjar, Kyle R Padgett, David Asher, Radka Stoyanova, John C Ford, Eric A Mellon

**Affiliations:** 1 Department of Radiation Oncology, Sylvester Comprehensive Cancer Center, Miller School of Medicine, University of Miami

**Keywords:** image-guided radiotherapy, treatment plannning, glioblastoma, mri guidance

## Abstract

Radiation therapy (RT) plays a critical role in the treatment of glioblastoma. Studies of brain imaging during RT for glioblastoma have demonstrated changes in the brain during RT. However, frequent or daily utilization of standalone magnetic resonance imaging (MRI) scans during RT have limited feasibility. The recent release of the tri-cobalt-60 MRI-guided RT (MR-IGRT) device (ViewRay MRIdian, Cleveland, OH) allows for daily brain MRI for the RT setup. Daily MRI of three postoperative patients undergoing RT and temozolomide for glioblastoma over a six-week course allowed for the identification of changes to the cavity, edema, and visible tumor on a daily basis. The volumes and dimensions of the resection cavities, edema, and T2-hyperintense tumor were measured. A general trend of daily decreases in cavity measurements was observed in all patients. For the one patient with edema, a trend of daily increases followed by a trend of daily decreases were observed. These results suggest that daily MRI could be used for onboard resimulation and adaptive RT for future fluctuations in the sizes of brain tumors, cavities, or cystic components. This could improve tumor targeting and reduce RT of healthy brain tissue.

## Introduction

The current standard of care in newly diagnosed glioblastoma includes the use of fractionated radiation therapy (RT) to 60 Gy in two Gy fractions delivered over six weeks with concurrent and adjuvant temozolomide following maximal safe resection of the tumor. The definition of target volumes for treatment is more controversial. European Organization for Research and Treatment of Cancer guidelines consider the surgical cavity volume and residual tumor volume, while Radiation Therapy Oncology Group guidelines also add the volume of the surrounding edema [1].

Target volumes are typically generated from preoperative and/or postoperative imaging fused with a computed tomography (CT) scan taken during the simulation of the patient [2]. The volumes of the resection cavity, residual tumor, and cerebral edema often change following surgery and over the course of RT, potentially leading to treatment volumes that do not accurately target the area of disease [2-3]. Magnetic resonance imaging (MRI) scans during RT could be used to assess these changes and allow for RT modifications; however, feasibility and cost have limited the frequent utilization of MRI scans. Further, while cone beam CT is a common form of image-guided radiotherapy (IGRT), it is inadequate for visualizing tumor-related changes within the brain due to poor soft tissue contrast. Alternatively, online magnetic resonance image guided-radiotherapy (MR-IGRT) could allow for the visualization of these structural changes, as it provides daily MRIs during treatment [4].

In this case series, we evaluated the daily MR-IGRT images of three glioblastoma patients treated on an integrated MRI-RT system to determine whether changes in resection cavity volume and cerebral edema can be observed on daily MRI. To our knowledge, this is the first study that reports on monitoring daily changes to the volumes of totally resected glioblastoma cavities, cranial edema, or any other brain malignancy.

## Case presentation

All three patients had World Health Organization (WHO) grade 4 glioblastomas. Patients 1 and 2 had no additional brain lesions and no significant edema surrounding the resection cavity. Patient 3 had an additional unresected a T2-hyperintense, non-gadolinium enhancing focus of suspected disease in the contralateral frontal lobe and obvious edema surrounding the resection cavity. Patients 1 and 2 were initially treated with gross total resection (GTR) and Patient 3 underwent GTR of the dominant enhancing mass. Thereafter, the patients were treated on the tri-cobalt-60 MRI-guided radiotherapy system (ViewRay MRIdian, Cleveland, OH). The delivered dose to the resection cavities was 60 Gy in 30 fractions delivered five times per week and 54 Gy to the secondary focus of disease over the same treatment schedule. The gross tumor volume (GTV) was defined as the surgical cavity volume and the T2-hyperintense focus of suspected disease, the clinical target volume (CTV) included a further 2 cm, and the planning target volume (PTV) included a further 3 mm. Scans used for RT planning included a non-contrast CT taken at the time of simulation, MRI taken on the ViewRay MRIdian system at the time of simulation, and a post-operative diagnostic MRI. Patients were concurrently treated with temozolomide 75 mg/m^2^ daily during treatment. None of the patients used steroids during their treatment. Table 1 lists additional features of the patients and their treatment course.

**Table 1:** Patient Demographics and Radiation Therapy Treatment Information RT: radiation therapy

Patient	Age (years)	Gender	Location of Cavity	Days Between Surgery and Simulation	Days Between Simulation and RT	Days of RT	Side Effects During Treatment	Length of Follow-up (months)
1	66	Female	Left temporal	16	11	45	Nausea, radiation dermatitis	4
2	39	Male	Right frontotemporal	39	7	39	Headaches, skin erythema, mild xeropthalmia	3
3	22	Male	Right frontoparietal with surrounding edema	25	13	41	Anxiety, insomnia, headaches, forehead rash	1

Methodology for volume measurements

**Figure 1:**
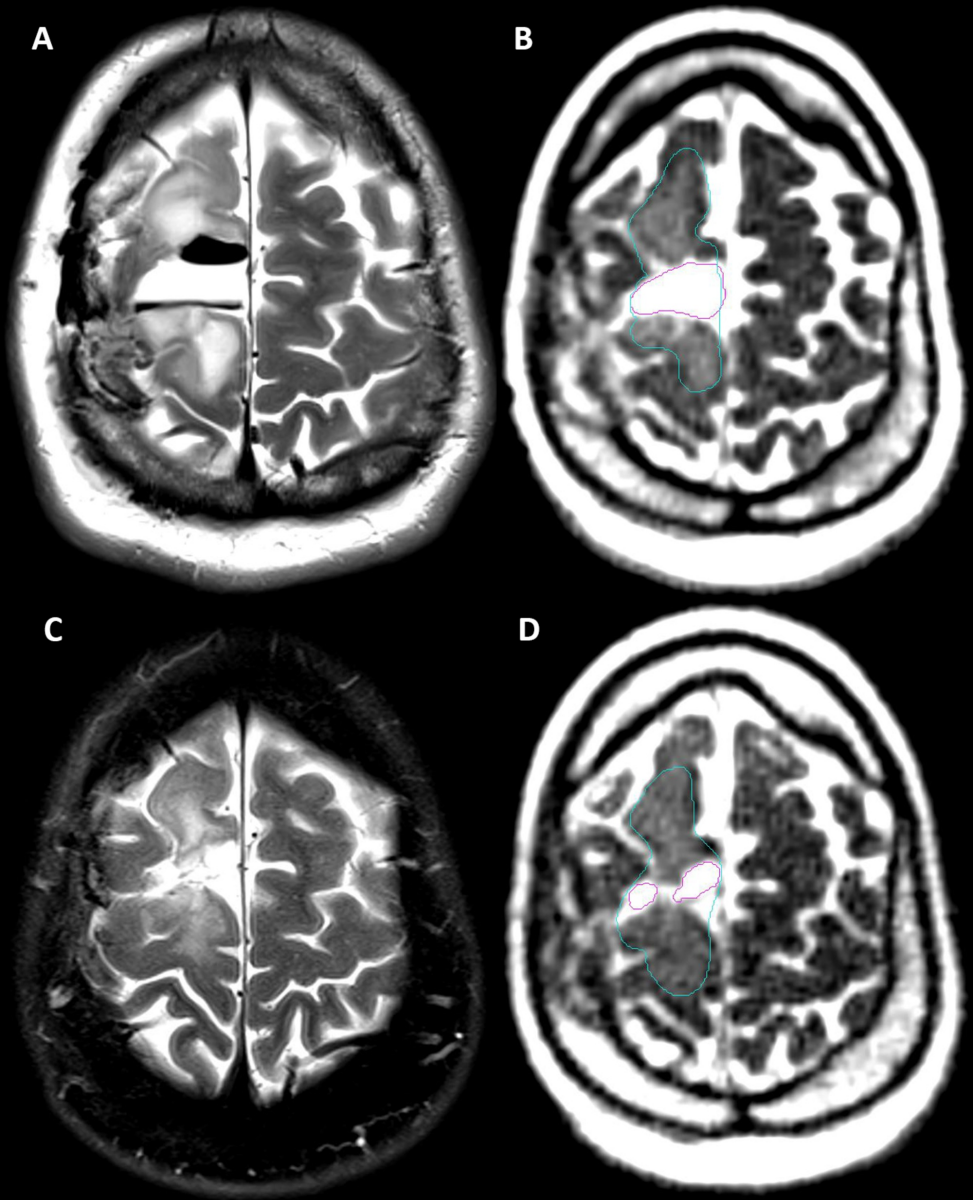
Comparison of Pre-radiation Therapy (RT) and Post-RT T2-weighted Magnetic Resonance Imaging (MRI) to Daily MRIs (A) Patient 3: T2-weighted MRI taken 23 days prior to simulation, showing T2-hyperintense resection cavity and edema with surrounding air pockets that were resorbed prior to simulation, (B) Patient 3: Simulation daily MRI showing a T2-hyperintense resection cavity (magenta) and edema (blue), (C) Patient 3: T2-weighted fat-saturated MRI taken 21 days after RT, showing a reduced T2-hyperintense resection cavity (magenta) and edema (blue), (D) Patient 3: Fraction 30 daily MRI showing a reduced T2-hyperintense resection cavity (magenta) and edema (blue) MRI: magnetic resonance imaging

Under an institutional review board (IRB) approved protocol, simulation and daily MRIs were obtained with the MR-IGRT system. Scans were performed with the balanced steady state free precession sequence (bSSFP, also known under the Siemens brand name TRUE-FISP or shortened to "TRUFI") included with the scanner (128 sec acquisition, 0.15 x 0.15 x 0.15 cm resolution, 40 x 43 x 40 cm field of view). This sequence acquires images that are a blend of T1 and T2, but there is dominant T2 weighting as evidenced by the bright cerebrospinal fluid, fat, and edema demonstrated in Figure 1. Resection cavities in all three patients as well as the edema and secondary tumor in Patient 3 were contoured by a single operator using MIM Software (MIM, Cleveland, OH). The volumes of these areas were generated from the contours. The cavity volume was not included in the volume measurement for edema in Patient 3. Edema was not contoured in Patients 1 and 2, as there was minimal edema on post-operative MRI and none or minimal edema seen on daily MR-IGRT. For the cavities and secondary tumor, the maximum axial diameter, the perpendicular axial diameter, and the maximum sagittal diameter were measured on the initial simulation daily MRI. The axial and sagittal dimensions of the resection cavities were measured on daily MRI of every fifth fraction on the same slice used in the initial measurement. Anatomical correlates were used to select a consistent image slice. For edema, the slices measured for the secondary tumor were arbitrarily selected and measured in a similar manner.

Results of volume measurements

**Figure 2:**
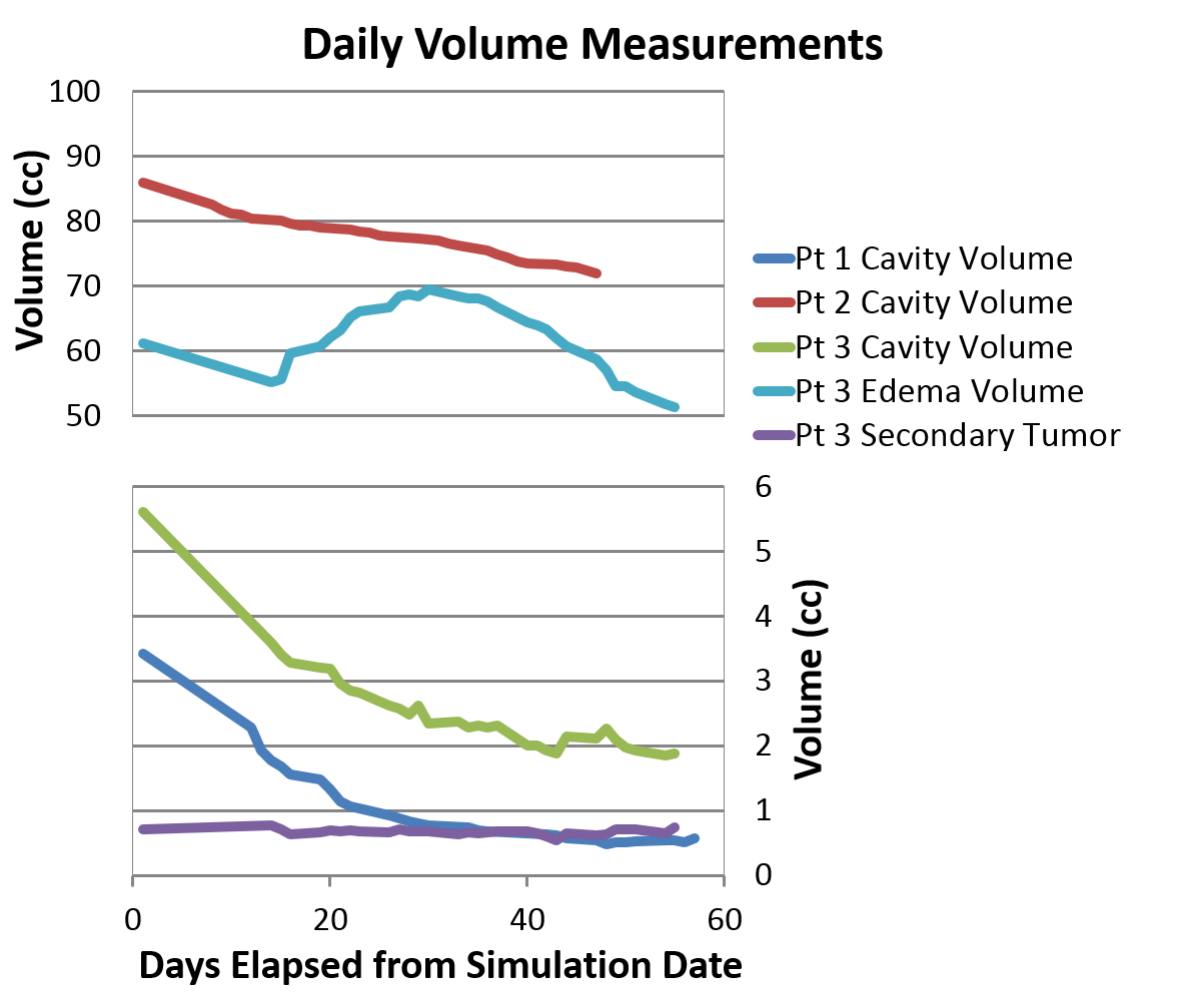
Trends of Simulation and Daily Fraction Volumes Pt: patient

**Figure 3:**
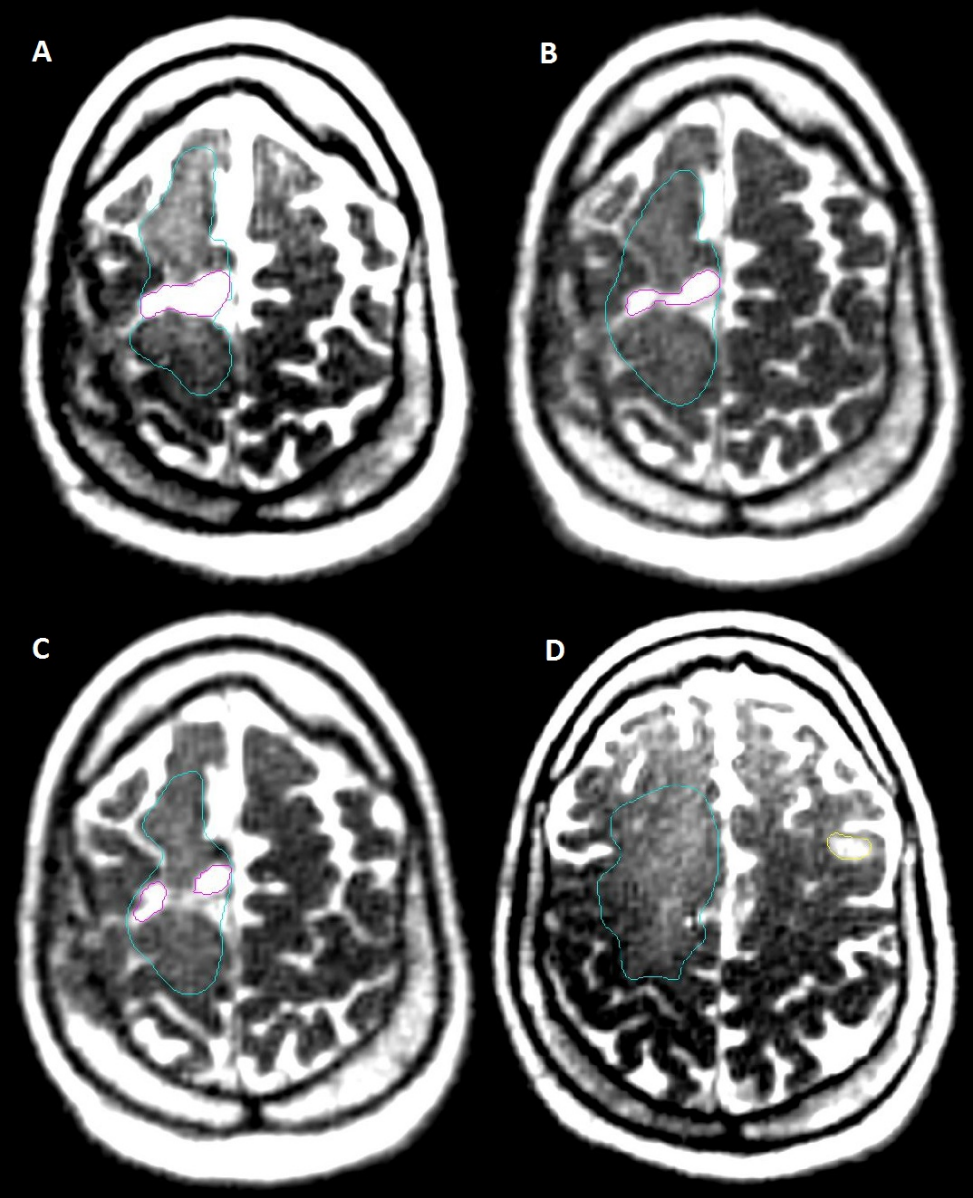
Patient 3: Cavity, Edema, and Secondary Tumor Simulation, Fraction 1, 15, 30 Daily Magnetic Resonance Imaging (MRI) (A) Patient 3: Fraction 1 containing a frontoparietal glioblastoma resection cavity (magenta) and surrounding edema (blue), (B) Patient 3: Fraction 15 showing that the cavity (magenta) has reduced in size slightly over the course of radiation therapy (RT) and the surrounding edema has increased, (C) Patient 3: Fraction 30 showing that the cavity has further reduced in size and the surrounding edema has decreased (D), Patient 3: Fraction 1 showing the secondary focus of disease (yellow) as well as edema (blue)

The daily volumes from simulation and the course of RT are graphed in Figure 2 and a summary of the overall difference in volumes over the course of simulation and RT is listed in Table 2. Figure 2 shows that the cavity volume for all three patients generally trended downward as time progressed. However, the cavity volume for Patients 1 and 3 gradually stabilized around Fraction 20 (day 43 and day 41, respectively). The volume of the secondary tumor seen in Patient 3 did not change significantly during the course of treatment. The volume of cerebral edema in Patient 3 decreased between the initial simulation MRI and the MRI taken at the start of treatment. As treatment continued, the volume of edema increased up until fraction 13 (day 30), at which point it began to decrease until the end of treatment. Figure 3 displays the daily MRIs of Patient 3.

**Table 2:** Measurement Changes over the Course of Radiation Therapy (RT)

		Simulation		Fraction 1		Fraction 15		Fraction 30	
Patient	Structure	Volume (cc)	Size (cm)	Volume (cc)	Size (cm)	Volume (cc)	Size (cm)	Volume (cc)	Size (cm)
1	Cavity	3.4	3.4x1.1x1.3	2.3	3.0x0.8x1.1	0.8	2.6x0.6x0.7	0.6	1.7x0.4x0.5
2	Cavity	86	7.4x3.5x8.1	82.6	7.2x3.3x8.0	77.6	7.1x3.1x7.6	72	6.9x3.0x7.5
3	Cavity	5.6	2.6x1.4x3.1	3.6	2.4x1.0x2.7	2.3	2.7x0.6x1.6	1.9	2.3x0.6x1.3
3	Secondary Tumor	0.7	1.4x0.6x1.2	0.8	1.2x0.6x1.1	0.7	1.3x0.7x1.1	0.7	1.3x0.7x1.2
3	Edema	61.2	6.8x4.1x8.0	55.2	5.7x3.6x7.4	68.1	6.2x3.6x6.8	51.4	5.8x2.8x5.8

## Discussion

This case series is the first report in the literature to monitor volume changes within the brains of patients undergoing radiotherapy on a daily basis. The study was successful in monitoring a general downward trend in cavity volume in all three patients and tracking the volume of edema in one patient, showing that it is feasible to track surgical resection cavity volumes and cerebral edema on a daily basis with online MR-IGRT. The cavity volume changes observed in this case series have been confirmed in a study, which retrospectively replanned treatment volumes on a CT scan taken five weeks after RT in 19 patients with glioblastoma treated with GTR. All but one patient had a reduction in GTV relative to the original planning GTV, showing that repeat imaging during RT and modification of RT volume reductions would have decreased the amount of brain tissue treated [5].

Additional studies have repeated imaging in patients with gliomas at various intervals and have come to similar conclusions supporting the use of imaging during the course of RT. Champ et al. noted significant changes to GTV and CTV between immediate postoperative MRI and MRI on the day of RT simulation among 24 patients with high-grade glioma [2]. Tsien et al. found GTV changes in 17 out of 19 patients with WHO grades 3 and 4 gliomas based on MRI at week three of RT [6]. Shukla et al. evaluated 15 patients with WHO grades 3 and 4 gliomas and found GTV decreased in 12 patients and GTV increased in three patients based on MRI at the end of week five of RT [7]. Finally, Yang et al. evaluated 11 patients with WHO grades 2, 3, and 4 gliomas and found decreases in the surgical cavity, GTV, and PTV in all patients based on CT and MRI taken at the end of RT [3].

Based on our results, online MR image guided-radiotherapy can address these well-documented changes in treatment volumes during the course of RT by allowing for replanning at regular intervals during RT. The proposed uses of online adapted MR-IGRT include reducing target volumes with cavity shrinkage, which could minimize the amount of healthy brain tissue receiving radiation. Additionally, brain tumors that grow during radiation, such as cystic components of craniopharyngiomas, could be seamlessly tracked and adapted during therapy [8]. Further, the daily monitoring of changes to cerebral edema may allow for the correlation of symptoms to cerebral edema and the monitoring of the responsiveness of cerebral edema to steroids.

The standard measures of glioblastoma response in use currently assess response at one or several months after the completion of radiotherapy. In addition to further anatomical assessments with additional brain sequences (T1, T2, gadolinium-enhanced T1, etc), the use of online MR-IGRT could allow for the assessment of tumor response and the adaption of therapy based on functional MRI changes (using advanced MRI techniques, such as diffusion or perfusion)—a process termed “functional adaptive radiotherapy.” For example, diffusion maps obtained during week three of RT predicts survival in glioblastoma and could allow for response-based therapy alteration [9]. The use of longitudinal diffusion MRI with the MR-IGRT system has already been deemed feasible in head and neck cancers and sarcomas to assess response to RT [10]. Future studies should implement longitudinal diffusion MRI on MR-IGRT systems for response-guided adaptive RT with high-grade gliomas.

## Conclusions

Past studies have demonstrated the potential benefits of regular MRI scans during the radiotherapy of brain tumors; however, the routine implementation of MRI has been limited by cost and feasibility. In this report, we have demonstrated that it is possible to delineate changes to brain tumor, cavity, and edema volumes with the images acquired for patient setup on a daily basis with MR-IGRT. Ultimately, these improvements could lead to enhanced treatment, reduced local recurrence of disease, and decreased radiation-induced neurotoxicity. Future studies should explore the clinical utility of daily adaptive MR-IGRT based on patient anatomy and the potential for functional adaptive MR-IGRT in gliomas. Additionally, the use of this technology in other central nervous system lesions with dynamic volumes should be investigated.
